# The sFlt-1/PlGF Ratio in Patients Affected by Gestational Diabetes and SARS-CoV-2 Infection

**DOI:** 10.3390/metabo13010054

**Published:** 2022-12-30

**Authors:** Daniela Denis Di Martino, Chiara Maria Soldavini, Gabriele Rossi, Maria Chiara Lonardoni, Gabriele Tinè, Agnese Caneschi, Francesco D’Ambrosi, Enrico Ferrazzi

**Affiliations:** 1 Obstetrics Unit, Department of Woman Child and Newborn, Fondazione IRCCS Ca’ Granda, Ospedale Maggiore Policlinico, 20122 Milan, Italy; 2 Department of Economics and Quantitative Methods, University of Milano Bicocca, 20900 Monza, Italy; 3 Department of Clinical and Community Sciences, University of Milan, 20122 Milan, Italy

**Keywords:** COVID-19, metabolic syndrome, biomarkers

## Abstract

Low values of the ratio of plasmatic soluble blocking factor FMS-like tyrosine Kinase 1 and placental growth factor (sFlt-1/PlGF) are required for an adequate placental angiogenesis and function. It has been shown that patients affected by gestational diabetes (GD) and patients with pneumonia from SARS-CoV-2 are characterized by an increased sFlt-1/PlGF ratio. The aim of the present study was to evaluate the sFlt-1-PlGF ratio in pregnancies complicated by COVID-19 and GD. We compared the plasmatic sFlt-1/PlGF ratio among the following groups of pregnant women: COVID-19, GD patients; COVID-19, non-GD patients; non-COVID-19, GD patients; and non-COVID-19, non-GD controls. We enrolled 62 women in the present study, who were divided as follows: 14 COVID-19, GD patients; 12 COVID-19, non-GD patients; 11 non-COVID-19, GD patients; and 25 non-COVID-19, non-GD controls. The COVID-19, GD patients presented a higher pre-pregnancy BMI, a higher prevalence of hypertensive disorders of pregnancy as a co-morbidity, and an increased need for medication for their diabetes. Neonatal data were similar between the groups. The controls showed a significantly lower sFlt-1/PlGF ratio compared to pregnancies complicated by GD and SARS-CoV-2 infection. The sFlt-1/PlGF ratio was higher in patients affected by both GD and SARS-CoV-2 infection; these subjects were characterized by a greater incidence of obesity and hypertensive disorders of pregnancy.

## 1. Introduction

Placental dysfunction (PD) is a pregnancy disorder that can lead to significant maternal-fetal complications, such as hypertensive disorders of pregnancy (HDP) or gestational diabetes (GD), which lead to maternal morbidity and mortality [1,2,3,4].

Normal placental development is characterized by a balanced maternal angiogenic/anti-angiogenic profile. In fact, during pregnancy, an appropriate evolution of placental growth factor (PlGF) and of its blocking factor (sFlt1) regulates a physiological villi angiogenesis and the remodeling of maternal spiral arteries [2].

Measurement of these factors and their ratio has become an increasingly useful diagnostic tool for the prediction of placental oxidative stress and of maternal endothelial damage [5,6].

During the pandemic period, in which SARS-CoV-2 infection was prevalent, physicians observed that this new infection was correlated with endothelial damage, especially in pregnant women [7,8]. Additionally, it is correlated with maternal comorbidities, with a predominant role being played by obesity and gestational diabetes, which are all proinflammatory conditions [9]. The link between these comorbidities and the infectivity and virulence of this virus is likely to be caused by glycosylation of the SARS-CoV-2 spike protein; this process may work as a glycan shield, facilitating immune evasion, as well as through spike and Angiotensin-Converting Enzyme 2 (ACE2) interactions [10]. This association between COVID-19 severity and the serum concentration of the soluble receptor for advanced glycation end-products (sRAGE) was proven in a cohort of 145 adult subjects by Zhao and coworkers [11]. Moreover, it has recently been shown that Angiotensin2-mediated endothelial dysfunction is associated with an increased sFlt-1/PlGF ratio in symptomatic patients with SARS-CoV-2 infections [12,13]; this may be due to the direct infection of the virus through the cellular entry receptor ACE2 and the state of general inflammation disrupting the equilibrium of the renin-angiotensin system pathway [7]. In addition, recent findings suggest an alteration in the placentation process through the interaction between the virus proteins and trophoblast cells [14].

The complex pathophysiology of SARS-CoV-2 infection includes immune-mediated effects in both pulmonary and extra-pulmonary tissues causing a state of general inflammation, which involves the hypercoagulability and up-regulation of the complement pathway caused by the virus, leading to either thrombotic or hemorrhagic manifestations [15]. When superimposed to pre-existing co-morbidity, the up-regulation of the immune system by SARS-CoV-2 infection puts pregnant patients at a major risk [12]. Since placental dysfunction may be expressed by a higher sFlt-1/PlGF ratio, this indicates that placental oxidative stress and SARS-CoV-2 infection and comorbidities such as gestational diabetes, obesity, and hypertensive disorders of pregnancy may increase inflammation and therefore worsen endothelial damage,

We hypothesized that pregnant patients with gestational diabetes present a higher level of sFlt-1/PlGF during SARS-CoV-2 infection. The aim of this study was to analyze sFlt-1 and PlGF concentrations and their ratio in patients positive for SARS-CoV-2 with gestational diabetes.

## 2. Materials and Methods

### 2.1. Study Population and Sample Collection

We conducted a prospective and comparative study using data from consecutive pregnant patients affected by SARS-CoV-2 infection who had been admitted to our COVID-19 maternity hub (Fondazione IRCCS Cà Granda Ospedale Maggiore Policlinico, Mangiagalli High Risk Maternity Centre, Milan, Lombardy, Italy) from February 2020 to May 2021.

These findings were compared with data from SARS-CoV-2-negative pregnant patients enrolled at admission to the same obstetrics unit or who attended obstetric visits at maternal-fetal medicine outpatient clinics from February 2020 to July 2021.

The study was approved by the Ethical Committee Milan Area 2 (Co-OST, n° 295_2021) and it was carried out in accordance with the Declaration of Helsinki.

Inclusion criteria were maternal age ≥ 18 years and gestational age > 24 weeks. Written formal consent was obtained from all the patients enrolled in the study. We excluded multiple pregnancies, fetal malformation, and other maternal conditions such as chronic kidney disease and infections during pregnancy (toxoplasma, cytomegalovirus, rubella, varicella zoster virus, hepatitis B and C virus, human immunodeficiency virus). Subjects on low-molecular-weight heparin therapy or prophylaxis prior to recruitment were not considered eligible for this study.

All patients received standard care for maternal and fetal well-being monitoring according to the protocols in use at our institute (assessment of vitals, obstetric ultrasound scans), as well as a nasopharyngeal swab and venous blood sampling for sFlt-1/PlGF ratio evaluation. SARS-CoV-2 infection was diagnosed by means of a positive nasopharyngeal swab obtained through PCR testing both in symptomatic and asymptomatic women. Gestational diabetes (GD) was diagnosed according to the recommendations of the International Association of Diabetes and Pregnancy Study Group (IADPSG) [16].

Patients were divided as follows: patients with gestational diabetes with SARS-CoV-2 (COVID-19, GD); patients with SARS-CoV-2 without any other pregnancy complications (COVID-19, non-GD); patients affected by gestational diabetes not affected by SARS-CoV-2 (non-COVID-19, GD); and pregnant women with uneventful pregnancies (controls). 

Patients diagnosed with GD underwent the protocol in use at our institute. All patients were initially treated with a tailored diet according to the reference intake levels of nutrients and energy for the Italian population (LARN) for pregnant women [17]. The dietary regimen included three main meals and two snacks with a balanced daily intake of macronutrients: carbohydrates (45% of total energy, less than 12% from simple sugar), fat (25–35% of total energy, less than 7% from saturated fat), and proteins (30–20% of total energy, 50% from animal source and 50% from vegetal source). Maternal pre-pregnancy BMI and weight gain were considered to tailor caloric intake.

The efficacy of diet alone was monitored by means of fetal growth assessment every two weeks and by the self-evaluation of pre- and post-prandial blood glucose levels, which were measured at regular weekly intervals. Fetal growth was measured every two weeks. Reference blood glucose levels were considered as follows: <95 mg/dL in fasting state, <120 mg/dL in 2 h post prandial state. When fetal growth in terms of abdominal circumference exceeded the 75th percentile according to local growth curves, the reference blood glucose levels were <90 mg/dL in the fasting state and <110 mg/dL in the 2 h post prandial state [18,19]. Pharmacological treatment for diabetes included metformin or insulin (long-acting at night, fast-acting at meals) or both. It was initiated when glucose levels were suboptimal (i.e., when 20% of the measurements or more were above reference values) or when abdominal fetal growth increased above the 75th percentile.

Venous blood samples for the analysis of sFlt-1 and PlGF concentrations were drawn at recruitment regardless of fasting and were collected in a vial containing a separating gel. Vials were centrifuged for 10 min within three hours of collection. sFlt-1 and PlGF concentrations were measured at recruitment by means of Elecsys automated systems (Roche Diagnostics, Rotkreuz, Switzerland); each electrochemiluminescence immunoassay required a sample volume of 20 µL for sFlt-1 and 50 µL for PlGF. Detection limits varied between 10 and 85,000 pg/mL for sFlt-1, and between 3 and 10,000 pg/mL for PlGF. The sFlt-1/PlGF ratio was generated according to these values.

Detailed information regarding demographics, clinical and laboratory data as well as treatment, obstetric, and neonatal outcome data were collected from electronic clinical records.

Among the demographic and clinical descriptors, we considered maternal age, pre-pregnancy BMI, gestational age at recruitment, the concurrent presence of hypertensive disorders of pregnancy (HDP) according to the International Society for the Study of Hypertension in Pregnancy criteria [20], and the need for admission to the intensive care unit (ICU). COVID-19 patients were classified according to symptoms and the need for oxygen respiratory support or mechanical ventilation. Mild infection was diagnosed in case of symptomatic infection (any of the following: temperature, cough, pharyngitis, rhinorrhea, ageusia, anosmia) without the need for oxygen support. Moderate disease referred to infection that needed oxygen support, while severe SARS-CoV-2 infection included the need for mechanical ventilation.

The collected obstetric and neonatal outcomes that were compared included gestational age at delivery, mode of delivery, neonatal weight (absolute values and percentiles calculated according to Italian weight charts for parity, gender, and gestational age in newborns [21]), and the rate of admission to the neonatal intensive care unit.

A sub-classification of diabetic patients was performed, according to the onset of late hypertensive disorders of pregnancy (above 32 weeks of gestation).

### 2.2. Statistical Analysis 

Non-parametric tests were used for the univariate analysis. Given the non-normal distribution, we used the Wilcoxon–Mann–Whitney U test for the comparison of the scalar variable between two groups; the Kruskall–Wallis test was used for multiple comparisons of the scalar variables; Fisher’s exact test was used for the categorical variables, in order to compare two groups; and the Fisher-Freeman-Halton exact test was used for multiple comparisons. Multivariate analysis was conducted including the sFlt-1/PlGF ratio as dependent variable and maternal pre-pregnancy BMI and maternal age as independent variables. Data were expressed by medians and interquartile ranges (IQRs) for scalar variables and with absolute and relative frequencies for categorical variables, respectively. 

No sample size was calculated for this pilot investigation, but the cases and controls were collected during the first and second waves of SARS-CoV-2 pandemic, which were characterized by a specific viral variant from Huang (China), that was more virulent than the modified ones that followed.

Statistical analysis of the data was performed using IBM SPSS Statistics software for Windows (version 23.0, IBM Corp., Armonk, NY, USA). A *p*-value of <0.05 was used as the limit of statistical significance.

## 3. Results

We enrolled 62 women and divided them into four groups: 14 COVID-19, GD patients; 12 COVID-19, non-GD patients; 11 non-COVID-19, GD patients; and 25 controls.

Table 1 reports the demographic and clinical data of these four groups. Older, but non-significant maternal age was observed in patients affected by GD (*p* = 0.06). Higher pre-pregnancy BMI and a higher rate of hypertensive disorders of pregnancy were significantly more present in GD patients and in diabetic COVID-19 patients compared to all of the other subjects. Moreover, the GD patients with COVID-19 required pharmacological treatment for diabetes more often than the pregnant women without the infection did; medications included insulin, metformin, or both. Most of the COVID-19 patients were asymptomatic (*n* = 5, 36% in the GD group, and *n* = 6, 50% in the non-GD group) or presented mild symptoms, with no need for O2 respiratory support or mechanical ventilation (*n* = 8, 57% in the GD group, and *n* = 4, 33% in the non-GD group). The following mild symptoms were considered: temperature, cough, pharyngitis, rhinorrhea, ageusia, and anosmia. The finding reported is confirmed by the low number of subjects admitted to intensive care unit (*n* = 1 GD patient and *n* = 1 non-GD patient). Five patients (19%) presented a diagnosis of pneumonia, 2 out of 14 (14%) in the GD group and 3 out of 12 (25%) in the non-GD group.

Table 2 reports the obstetric and neonatal outcomes of the four groups. We did not observe any significant differences between the groups for neonatal weight and for the proportion of new-born babies with weight above the 90° percentile. Diabetic patients affected by COVID-19 delivered at a median gestational age of 38 weeks, significantly earlier than the controls. The median new-born weight was adequate for the gestational age.

The distribution of values of the sFlt-1/PlGF ratio values in the four groups are shown in Table 3. Pregnancies complicated by both GD and SARS-CoV-2 infection only presented a significantly higher sFlt-1/PlGF ratio only when compared with the controls (*p* < 0.05). This was confirmed in the multivariate analysis that included maternal pre-pregnancy BMI and maternal age as independent variables (*p* = 0.008). None of the patients in the control group showed a sFlt-1/PlGF ratio greater than 38, while the sFlt-1/PlGF ratio was above 38 in four cases in 14 COVID-19, GD patients (29%), one case in 12 COVID-19, non-GD patients (8%), and one case of 11 non-COVID-19, GD patients (9%).

Table 4 reports and compares the values of sFlt-1, PlGF and the sFlt-1/PlGF ratio between diabetic subjects with COVID-19 according to the concurrent occurrence of hypertensive disorders. No significant differences were observed between the median values presented in these patients. The highest value of the sFlt-1/PlGF ratio was detected in one patient affected by hypertensive disorders of pregnancy (HDP), GD, and COVID-19; this value differs considerably from all of the other ones observed (sFlt-1/PlGF ratio 169; sFlt-1 9663 pg/mL; PlGF 57 pg/mL).

## 4. Discussion

To our knowledge, this is the first study exploring the level of the sFlt-1/PlGF ratio in pregnant patients affected by gestational diabetes and SARS-CoV-2 infection.

The main finding of this cohort of our study is the significantly higher sFlt-1/PlGF ratio in pregnant patients with COVID-19 and GD compared to the control group.

Oxidative stress is the result of the imbalance between pro-angiogenic and anti-angiogenic factors. This condition can be generated by increased inflammation, and can also determine further inflammation, resulting in a vicious circle that exacerbates the adverse environment. Part of the reported differences between the infected and non-infected women may be due to the intrinsic characteristics of the population. Indeed, the group of GD and COVID-19-positive patients presented a higher pre-gestational BMI and a high prevalence of HDP. These characteristics can contribute to the increase in the sFlt-1/PlGF. In a recent study by Nuzzo et al., the authors reported comparable sFlt-1/PlGF levels in pregnancies complicated by GD versus in the controls group [3]. Additionally, they found no differences in placental weight, birth weight, or in uterine and umbilical Doppler, thus confirming their hypothesis of a healthy fetal-placental unit in GD pregnancies. In addition, the same authors reported greater sFlt-1/PlGF values in GD when associated with pre-eclampsia, representing a sign of the increased syncytiotrophoblast oxidative stress.

Recently, an increased sFlt-1/PlGF ratio has also been observed in adult non-pregnant subjects COVID-19 with pneumonia compared to patients with COVID-19 without pneumonia, representing a marker of pulmonary endothelial oxidative stress [12]. Our results seem to confirm that SARS-CoV-2 infection poses a greater risk of a higher sFlt-1/PlGF ratio in pregnant patients with GD. The placental trophoblastic and the maternal pulmonary endothelial components of these findings cannot be easily disentangled. In fact, in pregnant patients with SARS-CoV-2 infection, this virus has been reported in more than 45% of placentas, as proven by immunochemistry, even without new-born infection [22].

Therefore, a severe COVID-19 infection could mimic pre-eclamptic conditions, suggesting an endothelial damage sustained by a significant increase in anti-angiogenic factors (sFlt-1) not mitigated by pro-angiogenic factors (PlGF).

In our study, a higher pre-pregnancy BMI and the incidence of hypertensive disorders of pregnancy were significantly more frequent in COVID-19 GD patients compared to in all other subjects. Maternal obesity and GD have been demonstrated to be associated with endothelial dysfunction [23,24]; this seems to be due to the abnormal expression and secretion of angiogenic proteins and adhesion molecules in omental adipose tissue [25]. Additionally, under this condition, adipocytokines dysregulation contributes to a pre-existing enhanced ACE2 expression and procoagulant state, thus leading to an increased susceptibility to SARS-CoV-2 infection and to more severe COVID-19 [9]. Obesity as risk factor for fetal and maternal morbidity has been confirmed in pregnant infected patients [26].

When the proinflammatory factors associated with the fat tissues of obese and diabetic patients are added to the AGE-RAGE axis, and when glycation enhances the virulence of the virus, a perfect storm could be hitting these patients.

### 4.1. Strengths and Limitations

All patients involved in the present study were recruited during the first and second waves of the SARS-CoV-2 pandemic, between March 2020 and May 2021, when pandemic hospital management was still being set up all around the world; these pandemic waves were characterized by a more virulent viral variant than the subsequent waves and by the absence of vaccine protection.

The main limitation of our findings is due to the limited number of patients enrolled; a larger sample will probably help to sort out the relative impact of these concurring co-factors. Moreover, although pharmacological treatments for COVID-19 might also have impacted the sFlt-1/PlGF level, none of the patients infected with SARS-CoV-2 had received low-molecular-weight heparin or specific anti-inflammatory drugs before the samples were collected.

### 4.2. Clinical and Research Implications of Our Findings

Although our study expanded the knowledge on endothelial dysfunction in GD complicated by SARS-CoV-2 infection, further investigation in a larger cohort is required to clarify the role of co-morbidities such as obesity and hypertensive disorders.

The prospective collection of samples from pregnant patients with SARS-CoV-2 infection is currently under way.

## 5. Conclusions

Our results show that the sFlt-1/PlGF ratio is increased in pregnant patients affected by GD and SARS-CoV-2 infection compared to controls, often in association with a higher body mass index and hypertensive disorders of pregnancy. In this group of patients, our findings reveal mild of oxidative stress, which is still not enough to alter neonatal outcomes. However, these data could suggest the possible role of sFlt-1/PlGF ratio analysis to monitor affected pregnant patients.

## Figures and Tables

**Table 1:** Maternal demographic and clinical data.

Variable	COVID-19, GD (*n* = 14)	COVID-19, Non-GD (*n* = 12)	Non-COVID-19, GD (*n* = 11)	Controls (*n* = 25)	*p*-Value (*)	Post Hoc Test
Maternal age (years)	34 (31–40)	28 (22–35)	33 (32–37)	32 (31–35)	0.06	
Pre-pregnancy BMI (kg/m^2^)	25 (23–35)	22 (22–25)	23 (21–25)	22 (19–26)	<0.02	§ #
Gestationl age recruitment (weeks)	36 (32–37)	34 (29–38)	35 (34–35)	35 (35–36)	0.64	
Medications for gestational diabetes	9 (64%)	-	1 (9%)	-	<0.03	*#*
HDP	5 (35%)	0 (0%)	0 (0%)	0 (0%)	<0.01	§ # †
Admission to ICU	1 (7%)	1 (8%)	0 (0%)	0 (0%)	0.35	
COVID-19						
Asymptomatic	5 (36%)	6 (50%)	-	-	0.69	
Mild-Moderate-Severe	9 (64%)	6 (50%)	-	-	0.69	

BMI, body mass index; HDP, hypertensive disorders of pregnancy; ICU, intensive care unit. (*) Kruskall–Wallis test or Fisher–Freeman–Halton exact test as appropriate. In post hoc test: § *p*-value < 0.05 for COVID-19, GD vs. COVID-19, non-GD; # *p*-value < 0.05 for COVID-19, GD vs. non-COVID-19, GD; † *p*-value < 0.05 for COVID-19, GD vs. controls.

**Table 2:** Mode of delivery and neonatal outcomes.

Variable	COVID-19, GD (*n* = 14)	COVID-19, Non-GD (*n* = 12)	Non-COVID-19, GD (*n* = 11)	Controls (*n* = 25)	*p*-Value (*)	Post Hoc Test
Gestational age at delivery (weeks)	38 (37–39)	39 (37–40)	39 (38–39)	39 (38–40)	0.03	†
Cesarean sections rate	8 (57%)	5 (41%)	4 (36%)	6 (24%)	0.22	
Neonatal weight (g)	3022 (2376–3402)	3175 (2837–3475)	3010 (2860–3535)	3280 (3140–3525)	0.19	
Neonatal weight > 90° percentile [21]	1 (7%)	2 (16%)	0 (0%)	3 (12%)	0.67	
Admission to NICU	2 (14%)	2 (16%)	0 (0%)	0 (0%)	0.08	

NICU, neonatal intensive care unit. (*) Kruskall–Wallis test or Fisher–Freeman–Halton exact test as appropriate. In post hoc test: † *p*-value < 0.05 for COVID-19, GD vs. controls.

**Table 3:** Values of soluble blocking factor FMS-like tyrosine Kinase 1 (sFlt-1), placental growth factor (PlGF) and the sFlt-1/PlGF ratio.

Value	COVID-19, GD (*n* = 14)	COVID-19, Non-GD (*n* = 12)	Non-COVID-19, GD (*n* = 11)	Controls (*n* = 25)	*p*-Value (*)	Post Hoc Test
sFlt-1	3721 (1601–6026)	1683.5 (1219–2386)	3544 (1479–4776)	2081 (1569–2970)	0.10	
PlGF	174 (114–454)	529 (149–953)	358 (204–754)	465 (282–618)	0.10	
sFlt-1/PlGF Ratio	26 (5–42.8)	6 (1.9–18.4)	8 (2.6–23.4)	5 (3–10)	0.047	†

(*) Kruskall–Wallis test. In post hoc test: † *p*-value < 0.05 for COVID-19, GD vs. controls.

**Table 4:** Values of soluble blocking factor FMS-like tyrosine Kinase 1 (sFlt-1), placental growth factor (PlGF), and the sFlt-1/PlGF ratio in COVID-19 positive diabetic patients with pregnancies complicated by late hypertensive disorders.

Variable	COVID-19, GD, with Late HDP (*n* = 5)	COVID-19, GD, without Late HDP (*n* = 9)	*p*-Value (*)
sFlt-1	432 (2508–9670)	350 (1569–5951)	0.36
PlGF	14 (97–465)	20 (104–446)	1.00
sFlt-1/PlGF Ratio	26 (12–100)	27 (5–44)	0.90
sFlt-1/PlGF Ratio >38	1 (20%)	3 (33%)	n.a.

HDP, hypertensive disorders of pregnancy; n.a., not assessed. (*) Wilcoxon–Mann–Whitney U test and Fisher’s exact test as appropriate.

## Data Availability

Data are unavailable since they are property of Fondazione IRCCS Ca’ Granda, Ospedale Maggiore Policlinico of Milan.

## References

[1] Redman C.W.G., Staff A.C., Roberts J.M. (2020). Syncytiotrophoblast stress in preeclampsia: The convergence point for multiple pathways. Am. J. Obstet. Gynecol..

[2] Silasi M., Cohen B., Karumanchi S.A., Rana S. (2010). Abnormal placentation, angiogenic factors, and the pathogenesis of preeclampsia. Obstet. Gynecol. Clin. N. Am..

[3] Nuzzo A.M., Giuffrida D., Moretti L., Re P., Grassi G., Menato G., Rolfo A. (2021). Placental and maternal sFlt1/PlGF expression in gestational diabetes mellitus. Sci. Rep..

[4] Zen M., Padmanabhan S., Zhang K., Kirby A., Cheung N.W., Lee V.W., Alahakoon T.I. (2020). Urinary and Serum Angiogenic Markers in Women with Preexisting Diabetes During Pregnancy and Their Role in Preeclampsia Prediction. Diabetes Care.

[5] Levine R.J., Maynard S.E., Qian C., Lim K.H., England L.J., Yu K.F., Schisterman E.F., Thadhani R., Sachs B.P., Epstein F.H. (2004). Circulating angiogenic factors and the risk of preeclampsia. N. Engl. J. Med..

[6] Verlohren S., Brennecke S.P., Galindo A., Karumanchi S.A., Mirkovic L.B., Schlembach D., Stepan H., Vatish M., Zeisler H., Rana S. (2022). Clinical interpretation and implementation of the sFlt-1/PlGF ratio in the prediction, diagnosis and management of preeclampsia. Pregnancy Hypertens..

[7] Verma S., Joshi C.S., Silverstein R.B., He M., Carter E.B., Mysorekar I.U. (2021). SARS-CoV-2 colonization of maternal and fetal cells of the human placenta promotes alteration of local renin-angiotensin system. Med.

[8] Oltean I., Tran J., Lawrence S., Ruschkowski B.A., Zeng N., Bardwell C., Nasr Y., de Nanassy J., El Demellawy D. (2021). Impact of SARS-CoV-2 on the clinical outcomes and placental pathology of pregnant women and their infants: A systematic review. Heliyon.

[9] Yanai H. (2020). Metabolic Syndrome and COVID-19. Cardiol. Res..

[10] Di Martino D., Cappelletti M., Tondo M., Basello K., Garbin C., Speciani A., Ferrazzi E. (2022). Glycation driven inflammation: COVID 19 severity in pregnant women and perinatal outcome. Nutrients.

[11] Zhao P., Praissman J.L., Grant O.C., Cai Y., Xiao T., Rosenbalm K.E., Aoki K., Kellman B.P., Bridger R., Barouch D.H. (2020). Virus-Receptor Interactions of Glycosylated SARS-CoV-2 Spike and Human ACE2 Receptor. Cell Host Microbe.

[12] Giardini V., Carrer A., Casati M., Contro E., Vergani P., Gambacorti-Passerini C. (2020). Increased sFLT-1/PlGF ratio in COVID-19: A novel link to angiotensin II-mediated endothelial dysfunction. Am. J. Hematol..

[13] Soldavini C.M., Di Martino D., Sabattini E., Ornaghi S., Sterpi V., Erra R., Invernizzi F., Tine G., Giardini V., Vergani P. (2022). sFlt-1/PlGF ratio in hypertensive disorders of pregnancy in patients affected by COVID-19. Pregnancy Hypertens..

[14] Seethy A.A., Singh S., Mukherjee I., Pethusamy K., Purkayastha K., Sharma J.B., Sharma R.S., Dhar R., Karmakar S. (2021). Potential SARS-CoV-2 interactions with proteins involved in trophoblast functions—An in-silico study. Placenta.

[15] Villar J., Ariff S., Gunier R.B., Thiruvengadam R., Rauch S., Kholin A., Roggero P., Prefumo F., do Vale M.S., Cardona-Perez J.A. (2021). Maternal and Neonatal Morbidity and Mortality Among Pregnant Women With and Without COVID-19 Infection: The INTERCOVID Multinational Cohort Study. JAMA Pediatr..

[16] International Association of Diabetes and Pregnancy Study Groups Consensus Panel (2010). International association of diabetes and pregnancy study groups recommendations on the diagnosis and classification of hyperglycemia in pregnancy. Diabetes Care.

[17] Italian Society of Human Nutrition (2014). Reference Intake Levels of Nutrients and Energy for the Italian Population (LARN).

[18] Rossi G., Somigliana E., Moschetta M., Bottani B., Barbieri M., Vignali M. (2000). Adequate timing of fetal ultrasound to guide metabolic therapy in mild gestational diabetes mellitus. Results from a randomized study. Acta Obstet. Gynecol. Scand..

[19] Bonomo M., Cetin I., Pisoni M.P., Faden D., Mion E., Taricco E., Nobile de Santis M., Radaelli T., Motta G., Costa M. (2004). Flexible treatment of gestational diabetes modulated on ultrasound evaluation of intrauterine growth: A controlled randomized clinical trial. Diabetes Metab..

[20] Brown M.A., Magee L.A., Kenny L.C., Karumanchi S.A., McCarthy F.P., Saito S., Hall D.R., Warren C.E., Adoyi G., Ishaku S. (2018). Hypertensive Disorders of Pregnancy: ISSHP Classification, Diagnosis, and Management Recommendations for International Practice. Hypertension.

[21] Bertino E., Spada E., Occhi L., Coscia A., Giuliani F., Gagliardi L., Gilli G., Bona G., Fabris C., De Curtis M. (2010). Neonatal Anthropometric Charts: The Italian neonatal study compared with other European studies. JPGN.

[22] Cribiù F.M., Erra R., Pugni L., Rubio-Perez C., Alonso L., Simonetti S., Croci G.A., Serna G., Ronchi A., Pietrasanta C. (2021). Severe SARS-CoV-2 placenta infection can impact neonatal outcome in the absence of vertical transmission. J. Clin. Investig..

[23] Dubova E.A., Pavlov K.A., Borovkova E.I., Bayramova M.A., Makarov I.O., Shchegolev A.I. (2011). Vascular endothelial growth factor and its receptors in the placenta of pregnant women with obesity. Bull. Exp. Biol. Med..

[24] Mordwinkin N.M., Ouzounian J.G., Yedigarova L., Montoro M.N., Louie S.G., Rodgers K.E. (2013). Alteration of endothelial function markers in women with gestational diabetes and their fetuses. J. Matern. Fetal Neonatal Med..

[25] Lappas M. (2014). Markers of endothelial cell dysfunction are increased in human omental adipose tissue from women with pre-existing maternal obesity and gestational diabetes. Metabolism.

[26] Di Martino D., Chiaffarino F., Patanè L., Prefumo F., Vergani P., Ornaghi S., Savasi V., Spinillo A., Cromi A., D’Ambrosi F. (2021). Assessing risk factors for severe forms of COVID-19 in a pregnant population: A clinical series from Lombardy, Italy. Int. J. Gynaecol. Obstet..

